# Molecular Investigation of Lymph Nodes in Colon Cancer Patients Using One-Step Nucleic Acid Amplification (OSNA): A New Road to Better Staging?

**DOI:** 10.1002/cncr.27667

**Published:** 2012-06-08

**Authors:** Ulrich Güller, Andreas Zettl, Mathias Worni, Igor Langer, Daniela Cabalzar-Wondberg, Carsten T Viehl, Nicolas Demartines, Markus Zuber

**Affiliations:** 1 Department of Medical Oncology and Hematology, Cantonal Hospital St. GallenSt. Gallen, Switzerland; 2 Department of Visceral Surgery and Medicine, University of BerneBerne, Switzerland; 3 Pathology ViollierBasel, Switzerland; 4 Department of Surgery, Lindenhof HospitalBerne, Switzerland; 5 Department of Surgery, Cantonal Hospital OltenOlten, Switzerland; 6 Department of Surgery, University Hospital BaselBasel, Switzerland; 7 Department of Surgery, Vaudois University Hospital CenterLausanne, Switzerland

**Keywords:** one-step nucleic acid amplification, colon cancer, staging, lymph node, histopathology

## Abstract

**BACKGROUND:**

A new diagnostic system, called one-step nucleic acid amplification (OSNA), has recently been designed to detect cytokeratin 19 mRNA as a surrogate for lymph node metastases. The objective of this prospective investigation was to compare the performance of OSNA with both standard hematoxylin and eosin (H&E) analysis and intensive histopathology in the detection of colon cancer lymph node metastases.

**METHODS:**

In total, 313 lymph nodes from 22 consecutive patients with stage I, II, and III colon cancer were assessed. Half of each lymph node was analyzed initially by H&E followed by an intensive histologic workup (5 levels of H&E and immunohistochemistry analyses, the gold standard for the assessment of sensitivity/specificity of OSNA), and the other half was analyzed using OSNA.

**RESULTS:**

OSNA was more sensitive in detecting small lymph node tumor infiltrates compared with H&E (11 results were OSNA positive/H&E negative). Compared with intensive histopathology, OSNA had 94.5% sensitivity, 97.6% specificity, and a concordance rate of 97.1%. OSNA resulted in an upstaging of 2 of 13 patients (15.3%) with lymph node-negative colon cancer after standard H&E examination.

**CONCLUSIONS:**

OSNA appeared to be a powerful and promising molecular tool for the detection of lymph node metastases in patients with colon cancer. OSNA had similar performance in the detection of lymph node metastases compared with intensive histopathologic investigations and appeared to be superior to standard histology with H&E. Most important, the authors concluded that OSNA may lead to a potential upstaging of >15% of patients with colon cancer. Cancer 2012. © 2012 American Cancer Society.

## INTRODUCTION

Approximately 20% to 25% of patients with lymph node-negative colon cancer will suffer from recurrent disease within 5 years.^1^-^3^ This phenomenon is explained in part by small lymph node tumor infiltrates, which remain undetected using current histopathologic workup.^4^, ^5^ Most commonly, only 1 level of hematoxylin and eosin (H&E) staining is performed to assess lymph nodes in patients with colon cancer. However, this approach analyzes only a very small and arbitrarily chosen part of the entire lymph node tissue and, thus, missing small tumor metastases is unavoidable. The suboptimal sensitivity of H&E staining for the detection of lymph node metastases in patients with colorectal cancer is well known.^4^-^7^ The lack of an impeccable detection method of lymph node metastases remains a very unsettling problem for the oncologist, because potentially under-staged patients may not receive beneficial adjuvant chemotherapy and, thus, will have a greater risk of local and distal recurrences as well as worse overall survival.^8^, ^9^

Compared with standard H&E staining of lymph nodes in patients with colon cancer, it has been demonstrated that multilevel sectioning and the use of immunohistochemistry (IHC) can improve the detection of small tumor infiltrates in lymph nodes.^4^, ^10^ Recently, a new diagnostic, semiautomatic system, called *one-step nucleic acid amplification* (OSNA), was developed for the potential detection of lymph node metastases. OSNA is based on reverse transcription–loop-mediated isothermal amplification (RT-LAMP)^11^ to amplify cytokeratin 19 (CK19) mRNA. CK19 is an epithelial marker, which, when identified in lymph nodes from patients with colorectal cancer, is highly suggestive of the presence of lymph node metastases.^12^-^14^ OSNA was used previously for the detection of breast cancer^12^-^17^ and gastric cancer^18^; however, currently, the data are very limited regarding the detection of colon cancer lymph node metastases.^19^, ^20^

The objective of the current investigation was to compare the performance of OSNA versus both standard H&E and intensive histopathologic analyses in the detection of colon cancer lymph node metastases. Our hypothesis was that OSNA would provide better lymph node staging in patients with colon cancer versus the current standard (conventional H&E analysis) and would have similar sensitivity and specificity for the detection of lymph node metastases compared with intensive histopathologic analysis using multilevel sectioning and IHC.

## MATERIALS AND METHODS

### Patients

This study involved 22 consecutive patients with stage I through III colon cancer. Patients with metastatic disease were excluded from the study.

### Study Design

The objective of the current prospective investigation was to compare the performance of OSNA with the performance of both standard H&E and intensive histopathologic analysis (multilevel sectioning and IHC) in the detection of colon cancer lymph node metastases. An intensive histologic workup (H&E plus IHC), as described below, was considered the gold standard for assessing the sensitivity and specificity of OSNA.

All included patients provided written informed consent. The study was approved by the local ethical committee and was compliant with the Declaration of Helsinki.

### Analysis of Lymph Nodes

After patients underwent tumor resection, a pathologist (A.Z.) who was present in the operating room received and immediately processed the native specimen. Lymph nodes were meticulously harvested from the pericolic fatty tissue. Following the study protocol, lymph nodes >3 mm in greatest dimension were included into the study for OSNA analysis as well as multilevel sectioning and IHC. Lymph nodes were cut into 1-mm slices by a cutter that was provided by Sysmex (Kobe, Japan). The slices for OSNA were shock-frozen in liquid nitrogen and stored at −70°C until molecular analysis was performed. The slices for histopathologic workup were fixed in formalin. All lymph nodes that measured <3 mm in greatest dimension were completely embedded for conventional histopathologic workup. The cutoff size of 3 mm was chosen for technical reasons: If the lymph nodes are smaller than 3 mm, then it is almost impossible technically to slice them into 4 pieces and perform further analyses as we did in the current study.

The analysis of lymph nodes was described in detail in the study protocol. Each lymph node was analyzed separately, cut into 4 slices, and continuously labeled *a* through *d*. Slices *a* and *c* were pooled together and analyzed using OSNA, and slices *b* and *d* were subjected to an intensive histologic workup (H&E and CK 19 IHC staining on 5 levels for each of the 2 lymph node slices).

### Intensive Histologic Work-Up

Slices *b* and *d* were fixed with neutral buffered formaldehyde and processed on paraffin blocks. One initial level and additional levels with a 0.20-mm skip space were cut from slices *b* and *d* until no remnant remained. From the initial level and from the subsequent 4 levels, four 4-μm sections were used for H&E staining and CK19 IHC (DAKO, Glostrup, Denmark; 1:400 dilution). Thus, 5 H&E sections and 5 CK19 IHC sections were systematically prepared from each block. CK19 was chosen as the target marker because it is both highly specific and sensitive for the detection of colorectal cancer lymph node metastases. In a recent publication by Yamamoto et al, CK19 was expressed in all 85 colorectal cancer specimens, and most specimens (94%) had high expression.^20^ On the basis of this strong expression of CK19, the use of other markers was omitted, because this would have increased the costs and potentially would have decreased the specificity of the assay (producing a higher rate of false-positive results). According to the study protocol, lymph nodes that harbored isolated tumor cells (≤0.2 mm in greatest dimension) were considered negative.

### One-Step Nucleic Acid Amplification

The OSNA method is based on the detection of CK19 mRNA as a marker for colorectal cancer cells. A cutoff value of 250 mRNA copies/μL was used in the current investigation. A result with a CK19 mRNA copy number <250/μL was regarded as negative, and a copy number ≥250/μL was considered a positive result.

There is an association between the number of tumor cells in the specimen and the OSNA result: the more tumor cells present in a specimen, the faster the predetermined threshold of positivity will be reached.^13^, ^20^ The amount of tumor tissue present in the specimen is determined by the rise time, eg, the time to exceed a predetermined threshold of turbidity, which is caused by magnesium pyrophosphate, a by-product that is released during the amplification. The higher the tumor load in the specimen, the shorter the rise time will be and, thus, the faster the threshold of turbidity will be reached.^13^, ^20^

The determination of the cutoff level between positive and negative results was described in and based on the publications by Tsujimoto et al^13^ and Yamamoto et al.^20^ However, this cutoff level was established to detect micrometastases and macrometastases, while isolated tumor cells fall below the threshold of sensitivity. Rapid mRNA detection was achieved by homogenizing slices of the dissected lymph nodes in 4 mL homogenizing buffer (Lynorhag; Sysmex, Kobe, Japan) and directly amplifying CK19 mRNA without prior extraction or purification of nucleic acids (DNA and/or RNA). Lysates were prepared according to the standard operating procedure of the manufacturer. OSNA analysis was performed with the ready-to-use Lynoamp reaction kit on the RD-100i (Sysmex). Approximately 40 to 45 minutes were used for the investigation of 3 or 4 lymph nodes, including preparation and amplification time. To avoid observer bias, the OSNA analyses were done in a blinded fashion without knowledge of findings from the intensive histologic workup.

### Investigation of Discordant Cases

All discordant cases (OSNA-positive/histology-negative or OSNA-negative/histology-positive) were analyzed further with quantitative reverse-transcription-polymerase chain reaction (qRT-PCR) using CK19 and carcinoembryonic antigen (CEA) as described elsewhere.^19^ If the qRT-PCR confirmed the OSNA result, then we concluded that discordant findings were based on tissue allocation bias. This means that the differences between the intensive histologic workup and the OSNA analysis were because some small tumor infiltrates were localized only in the half of the lymph node that was analyzed either by OSNA or by histology. Therefore, these cases were excluded from the final analysis, because a comparison of the 2 methods was not feasible.

## RESULTS

In this prospective study, 313 lymph nodes from 22 consecutive patients with stage I to III colon cancer were analyzed. There were 10 women and 12 men, and the median patient age was 76 years (range, 55-88 years). The median number of harvested lymph nodes per patient was 30 (range, 16-60 lymph nodes), and a median of 13 lymph nodes (range, 6-24 lymph nodes) were analyzed using OSNA. Grading, tumor size, and tumor stage are provided in Table 1.

**Table 1:** Tumor Description

Variable	No. of Patients (%)
Total	22 (100)
Grade	
1: Well differentiated	0 (0)
2: Moderately well differentiated	18 (81.8)
3: Poorly differentiated	4 (18.2)
4: Undifferentiated	0 (0)
Pathologic tumor classification	
pT1	0 (0)
pT2	7 (31.8)
pT3	12 (54.5)
pT4	3 (13.6)
Stage	
I	6 (27.3)
II	7 (31.8)
III	9 (40.9)

Fifty-one lymph nodes were positive and 246 lymph nodes negative with both OSNA and standard H&E (Table 2). OSNA was more sensitive for detecting small tumor infiltrates compared with H&E (11 OSNA-positive/H&E-negative) (Table 2). Of those 11 lymph nodes, 1 harbored a micrometastasis, 5 harbored isolated tumor cells, and 5 remained negative after multilevel sectioning and IHC (Tables 2, 3).

**Table 2:** Comparison of One-Step Nucleic Acid Amplification With Standard Hematoxylin and Eosin Analysis (n = 313)

	Histology: First-Level H&E			
	Positive		Negative	
OSNA	Macrometastasis	Micrometastasis	ITC	No ITC
Positive^a^	51	0	0	11^b^
Negative	2^b^	0	3	246

Abbreviations: H&E, hematoxylin and eosin; ITC, isolated tumor cells; OSNA, one-step nucleic acid amplification.

a OSNA positivity is defined as >250 cytokeratin 19 mRNA copies/μL.

b Discordant cases.

**Table 3:** Comparison of One-Step Nucleic Acid Amplification With Intensive Histologic Workup (n = 313)

	Histology: H&E and IHC^a^			
	Positive		Negative	
OSNA	Macrometastasis	Micrometastasis	ITC	No ITC
Positive^b^	51	1	5^c^	5^c^
Negative	2^c^	3^c^	77	169

Abbreviations: H&E, hematoxylin and eosin; IHC, immunohistochemistry; ITC, isolated tumor cells; OSNA, one-step nucleic acid amplification.

a Two lymph node slices with 5 levels each were analyzed with H&E and IHC.

b OSNA positivity is defined as >250 cytokeratin 19 mRNA copies/μL.

c Discordant cases.

In 77 lymph nodes that were negative according to OSNA, isolated tumor cells were identified using intensive histopathologic workup (Table 3). When OSNA was compared with intensive histopathologic workup, there were 15 discordant cases (10 OSNA-positive/histology-negative, 5 OSNA-negative/histology-positive). All discordant cases were analyzed further using qRT-PCR for CK19 and CEA. In 6 cases (4 OSNA-positive/histology-negative, 2 OSNA-negative/histology-positive), the discordant case investigation using qRT-PCR confirmed the OSNA result; thus, we concluded that these 6 discordant findings were based on tissue allocation bias (eg, tumor infiltrates were present exclusively in the half of the lymph node analyzed either by OSNA or by histology) (Table 4). After excluding these 6 cases and comparing the results from an intensive histopathologic workup, OSNA had a sensitivity of 94.5% and a specificity of 97.6% to detect lymph node metastases with a concordance rate of 97.1%.

**Table 4:** Comparison of One-Step Nucleic Acid Amplification With Intensive Histologic Workup After Discordant Case Investigation (n = 307)

	Histology: H&E and IHC^a^			
	Positive		Negative	
OSNA	Macrometastasis	Micrometastasis	ITC	No ITC
Positive^b^	51	1	4^c^	2^c^
Negative	1^c^	2^c^	77	169

Abbreviations: H&E, hematoxylin and eosin; IHC, immunohistochemistry; ITC, isolated tumor cells; OSNA, one-step nucleic acid amplification.

a Two lymph nodes slices with 5 levels each were analyzed with H&E and IHC.

b OSNA positivity is defined as >250 cytokeratin 19 mRNA copies/μL.

c Discordant cases.

Two initially lymph node-negative patients were upstaged by OSNA (2 of 13 patients; 15.4%). One patient who had a positive OSNA result (620 copies/μL) had a micrometastasis identified only after multilevel sectioning and IHC, whereas the initial H&E examination was negative. The other patient who was upstaged had a strongly positive OSNA evaluation (1800 copies/μL). This finding was confirmed with qRT-PCR results that were positive for both CK19 and CEA. However, the histologic analysis was negative.

## DISCUSSION

On the basis of the current prospective study, OSNA appeared to be a new and powerful molecular tool for the detection of lymph node macrometastases and micrometastases in our series of 22 consecutive patients with colon cancer. The performance of OSNA was similar to that of intensive histopathologic investigations and appeared to be superior to current H&E analysis. Because OSNA allows an analysis of the entire lymph node, the often observed problem of sampling bias because of insufficiently analyzed material in standard H&E analyses can be overcome in the future. Therefore, OSNA may improve the staging of patients with colon cancer through the detection of otherwise hidden tumor deposits.

A relevant proportion of patients who have lymph node-negative colon cancer will develop local recurrences.^1^-^3^, ^21^ One contributing factor to this recurrence rate is the lack of detection of small lymph node metastases using the current H&E-based histopathologic assessment.^7^, ^22^ The current National Comprehensive Cancer Network (NCCN) recommends adjuvant chemotherapy for all patients with lymph node-positive disease.^23^ Therefore, it is crucial to identify all patients who have positive lymph nodes to decrease the risk of local and distant failure. In the current investigation, OSNA appeared to improve the detection of lymph node metastases. This improved staging method may lead to better patient selection for adjuvant chemotherapy and consecutively improved local and distant control as well as better overall survival.

The current study provides compelling evidence that OSNA is both very sensitive and specific for the assessment of lymph node metastases in patients with colon cancer and is comparable to extensive histopathologic workup using multilevel sectioning and IHC. Furthermore, it is crucial to bear in mind for the interpretation of this study that only half of each lymph node was assessed by OSNA, because it was essential to compare the new, investigational OSNA method with both standard H&E analyses and multilevel sectioning and IHC. The sensitivity of OSNA for the detection of small tumor infiltrates may be even greater when an entire lymph node is subjected to OSNA analysis. In OSNA-positive/histology-negative cases, which were identified as negative in subsequent qRT-PCR analyses (and, thus, were classified as false-positive), it is possible that the results actually were true-positive, because prolonged contact between the homogenizing buffer and the sample may have had a negative impact on the quality of mRNA, which subsequently may have been rendered undetectable.

In the current study, 1 macrometastasis remained undetected by OSNA (Table 4). This macrometastasis was largely necrotic, which explains the finding, because no mRNA was available for amplification in the OSNA analysis or qRT-PCR. However, as observed in our analysis, such findings are rare and usually observed in large, already macroscopically evident lymph node metastases that do not require any molecular workup.

Currently, there is no universal standard of histopathologic workup of lymph nodes in colorectal resection specimens that will assure the detection of most if not all embedded lymph node metastases. The latest guidelines of the College of American Pathologists recommend submitting all grossly negative or equivocal lymph nodes in their entirety, and routine assessment can be limited to conventional histologic techniques.^24^ Because the College of American Pathologists considers current data insufficient to recommend special measures for the detection of small tumor infiltrates, neither multiple levels of paraffin blocks nor the use of any ancillary techniques like IHC are recommended currently for the routine examination of lymph nodes.^25^

It appears evident that a simple analysis with H&E is insufficient for the assessment of lymph nodes in patients with colon cancer. It may be reasonable to argue that all lymph nodes should be analyzed using extensive histopathologic workup, including multilevel sectioning and IHC, to have the best possible staging method. However, this is a very time-consuming approach. Conversely, OSNA is a standardized, reproducible method and appears to be a sensitive and specific diagnostic tool for the detection of lymph node metastases in patients with colon cancer that can be performed semiautomatically.^19^, ^20^ Also, a major advantage of OSNA compared with qRT-PCR is that the latter requires RNA purification, whereas OSNA analysis can be done directly from the lysate. This results in a timely evaluation of the specimen using OSNA.

In the study by Yamamoto and colleagues,^20^ OSNA was compared with histopathologic examination in 385 lymph nodes from 85 patients who had colorectal cancer. Half of each lymph node was analyzed using OSNA, and the other half was subjected to histologic workup. The authors reported a high concordance rate between OSNA and histologic examination of 0.97. However, Yamamoto and colleagues did not use IHC as a gold standard but used only H&E examination in the analysis of those 385 lymph nodes. Conversely, in our prospective study, all lymph nodes were subjected to H&E analysis as well as IHC in the comparison with OSNA.

Recently, Croner et al published their findings on the use of OSNA to evaluate 184 lymph nodes from 184 patients with colorectal cancer.^19^ Similar to our investigation, those authors reported a high concordance rate between histology and OSNA (95.7%) for macrometastases and micrometastases. Also, both sensitivity and specificity were approximately 95% in their investigation. However, their report differs from the current study in many ways: First, the investigation by Croner et al was retrospective, whereas our study was entirely prospective. Second, whereas Croner et al used 1 randomly chosen lymph node for each patient with colorectal carcinoma, a median of 13 lymph nodes per patient were analyzed molecularly using OSNA in our study. Finally, surprisingly, Croner et al did not identify any lymph nodes with isolated tumor cells, which have been observed in up to 76% of immunohistochemically analyzed colorectal lymph nodes in most comparable studies.^2^, ^7^, ^26^

The choice of the cutoff level between a positive and negative OSNA result in the current study was based on the publications by Tsujimoto et al^13^ and Yamamoto et al.^20^ Yamamoto and colleagues used a cutoff value between positive and negative lymph nodes from patients with colorectal cancer based on the logarithmic midpoint between the maximum value of the CK19 mRNA copy number in lymph nodes from pN0 patients with 2 standard deviations from the average of CK19 mRNA copy number in histopathologically positive lymph nodes.^20^ In the study by Tsujimoto et al, the cutoff value for OSNA also was set at 250 CK19 mRNA copies/μL based on 84 histopathologically negative lymph nodes. The mean value of CK19 mRNA expression with 3 standard deviations amounted to 250 CK19 mRNA copies/μL.^13^ This cutoff level was established to detect micrometastases and macrometastases. In most publications that tested OSNA for the detection of lymph node micrometastases and macrometastases, the cutoff was set at 250 CK19 mRNA copies/μL.^13^, ^17^, ^19^, ^20^ Although some of those investigations were performed in patients with breast cancer, the results of the molecular analyses are transferable to the setting of patients with colon cancer. Therefore, the cutoff level of 250 copies of CK19 mRNA copies/μL also was chosen for the current investigation.

Although OSNA is very sensitive for the detection of colorectal cancer macrometastases and micrometastases, it must be emphasized that OSNA is not intended to detect isolated tumor cells. Indeed, the cutoff chosen for OSNA positivity (250 copies/μL) will not be reached if only isolated tumor cells are present, as clearly reflected in Table 4: Seventy-seven isolated tumor cells that were detected immunohistochemically were negative in OSNA. However, the prognostic implications of small tumor infiltrates in lymph nodes from patients with colon cancer are unknown and remain a matter of great debate.^5^, ^23^, ^27^-^30^

In the current study, 2 patients who had lymph node-negative results from an H&E analysis of 1 slice potentially were upstaged using OSNA (2 of 13 patients; 15.4%). However, 1 patient with a positive OSNA result and a negative initial H&E examination had a micrometastasis identified only after multilevel sectioning and IHC were performed. The second patient had a strongly positive OSNA result (1800 copies/μL). This positive finding was confirmed with qRT-PCR for both CK19 and CEA. The histologic analysis, however, was negative. It could be argued that this was a false-positive finding, because no tumor cells were detected using either H&E or IHC; however, the confirmation of the positive OSNA result by qRT-PCR analysis for both CEA and CK19 makes this highly unlikely. Moreover, this finding may be explained by tissue allocation bias, eg, the tumor cells were present only in the half of the lymph node that was analyzed by OSNA. Nonetheless, these results underline the importance and the potential of the OSNA assay as an improved staging method for patients with colon cancer, because the entire lymph node is analyzed with OSNA.

Still, we would like to highlight some difficulties applying OSNA to colon cancer specimens. First, OSNA currently relies on the analysis of fresh material. Although OSNA may be used on formalin-fixed material in the future, to date, this is not possible. Therefore, OSNA requires the immediate availability of a pathologist for lymph node harvesting, whereas formalin-fixed resection specimens can by processed at any time. Isolating lymph nodes from fresh tissue requires more time, diligence, and experience than when performed on well fixed resection specimens. However, the time spent for lymph node sampling will be more than compensated during the analytic process. Second, processing colorectal specimens for OSNA requires extreme care to avoid tissue contamination, a problem that usually can be resolved easily in conventional microscopy. Colorectal tumors can be large and friable; and, because CK19 is a panepithelial marker present in normal colonic epithelium and primary tumors, contamination of material for molecular analysis by dislodged tissue fragments during specimen processing has to be avoided. Third, care has to be taken to macroscopically distinguish lymph node metastasis from tumor spread into the pericolic fatty tissue. One possible solution to circumvent this problem would be to limit OSNA analysis to patients with clinically lymph node-negative tumors. Finally, a comparison of sensitivity for detecting micrometastases and macrometastases between OSNA and RT-PCR would be of interest. However, because the objective of the current study was to compare OSNA with both H&E and IHC, a further comparison between OSNA and RT-PCR was beyond the scope of this investigation but should be performed in future prospective studies.

In conclusion, based on the current prospective investigation, OSNA appears to be a powerful and promising new molecular tool for the detection of lymph node metastases in patients with colon cancer. The performance of OSNA in detecting micrometastases and macrometastases is similar to that of histopathologic investigations and appears to be superior to standard histopathologic workup with H&E. Because OSNA allows an analysis of the whole lymph node, the problem of sampling bias and undetected tumor deposits because of uninvestigated material will be overcome in the future. Therefore, OSNA may improve the staging of patients with colon cancer.

The interesting findings of our study should be corroborated in a randomized controlled trial. The current study indicates that patients with colon cancer should be randomized to standard H&E analysis of lymph nodes versus lymph node assessment using OSNA analysis. Although the current findings suggest that OSNA is superior to standard H&E for the assessment of lymph nodes in patients with colon cancer, a larger, prospective cohort study will be necessary to provide further evidence.
